# Postmastectomy radiation therapy for implant-based breast reconstruction: a systematic review and meta-analysis for the 2022 Japanese Breast Cancer Society Clinical Practice Guideline

**DOI:** 10.1007/s12282-025-01788-2

**Published:** 2025-09-30

**Authors:** Mami Ogita, Subaru Sawayanagi, Haruka Jinnouchi, Michio Yoshimura, Chikako Yamauchi, Naoko Sanuki, Yasushi Hamamoto, Kimiko Hirata, Mariko Kawamura, Yutaka Yamamoto, Shigehira Saji, Tatsuya Toyama

**Affiliations:** 1 Department of Radiology, The University of Tokyo Hospital, 7-3-1 Hongo, Bunkyo-Ku, Tokyo, 113-8655 Japan; 2 Department of Radiology, Tokyo Teishin Hospital, 2-14-23 Fujimi, Chiyoda-Ku, Tokyo, 102-8798 Japan; 3 Department of Radiation Oncology and Image-Applied Therapy, Graduate School of Medicine, Kyoto University, 54 Shogoin-Kawahara Cho, Sakyo-Ku, Kyoto, Kyoto 606 8507 Japan; 4 Department of Radiation Oncology, Shiga General Hospital, 5-4-30 Moriyama, Moriyama, Shiga 524-8524 Japan; 5 Department of Radiology, Keio University School of Medicine, 35 Shinanomachi, Shinjuku-Ku, Tokyo, 160-8582 Japan; 6 Department of Radiation Oncology, National Hospital Organization Shikoku Cancer Center, Ko-160, Minamiumemoto-Machi, Matsuyama, Ehime 791-0280 Japan; 7 Department of Radiation Therapy, Osaka Red Cross Hospital, 5-30 Fudegasakicho, Tennouji-Ku, Osaka, Osaka 543-8555 Japan; 8 Department of Radiology, Nagoya University Graduate School of Medicine, 65 Tsurumai-Cho, Showa-Ku, Nagoya, Aichi 466-8550 Japan; 9 Department of Breast and Endocrine Surgery, Graduate School of Medical Sciences, Kumamoto University, 1-1-1 Honjo, Chuo-Ku, Kumamoto, Kumamoto 860-8556 Japan; 10 Department of Medical Oncology, Fukushima Medical University, 1 Hikarigaoka, Fukushima, Fukushima 960-1295 Japan; 11 Department of Breast Surgery, Nagoya City University, 1 Kawasumi, Mizuho-Cho, Mizuho-Ku, Nagoya, Aichi 467-8601 Japan

**Keywords:** Breast cancer, Radiotherapy, Mammaplasty, Reconstruction, Implant

## Abstract

**Background:**

Implant-based breast reconstruction is the most commonly performed reconstructive technique following mastectomy. With an increasing number of patients undergoing implant-based breast reconstruction, concerns have arisen regarding the safety of postmastectomy radiation therapy (PMRT) in reconstructed breasts. This study aimed to investigate the safety of PMRT in implant-based breast reconstruction.

**Methods:**

A comprehensive literature search was conducted for articles published up to March 2021. Eligible studies included clinical trials and observational studies comparing outcomes between patients with breast cancer undergoing immediate implant-based breast reconstruction with PMRT and those without PMRT. The primary outcomes included major complications, reconstruction failure, capsular contracture, and cosmetic outcomes. Pooled odds ratio (OR) with 95% confidence interval (CI) were calculated using a random-effects model.

**Results:**

A total of 23 studies were identified, comprising one case–control study, one prospective cohort study, and 21 retrospective cohort studies. PMRT was significantly associated with increased rates of major complications (OR 2.62, 95% CI 1.82–3.77, *P* < 0.00001), reconstruction failure (OR 2.53, 95% CI 2.00–3.20, *P* < 0.00001), and capsular contracture (OR 9.63, 95% CI 5.77–16.06, *P* < 0.00001). Furthermore, cosmetic outcomes were significantly poorer in patients undergoing PMRT compared with those not receiving PMRT (OR 3.55, 95% CI 1.80–6.98, *P* < 0.003).

**Conclusions:**

This meta-analysis demonstrated that PMRT in implant-based breast reconstruction is associated with a significantly increased risk of adverse outcomes. Given these risks, treatment decisions should involve through discussions with patients to ensure that they are fully informed of the potential benefits and complications.

**Supplementary Information:**

The online version contains supplementary material available at 10.1007/s12282-025-01788-2.

## Introduction

Breast reconstruction after mastectomy plays an important role in breast cancer treatment. It offers psychological health benefits and higher patient satisfaction rates [1]. In recent years, the number of breast reconstruction procedures has increased [2]. According to the 2020 annual report of the National Clinical Database‑Breast Cancer Registry, among 48,040 patients who underwent mastectomy in Japan, 5,563 (11.6%) received breast reconstruction [3]. Although the percentage of patients opting for breast reconstruction remains relatively low, interest in breast reconstruction has grown, and the demand for accurate information continues to rise.

Implant-based prosthetic and autologous reconstructions are the major reconstructive options following mastectomy. The choice of reconstructive technique primarily depends on patient preference as well as clinical and physical factors. Implant-based breast reconstruction is often preferred due to its shorter operative and recovery times, relatively smaller incisions, and the absence of donor-site morbidity. In Japan, this method is the most widely used. In 2020, 66.4% of patients who underwent breast reconstruction after mastectomy chose implant-based reconstruction [3]. Despite its advantages and widespread used, implant-based reconstruction has limitations, including the eventual need for implant replacement due to their finite lifespan and a less natural feel [4, 5]. Additionally, complications, such as infection, capsular contracture, rupture, malposition, and breast implant-associated anaplastic large-cell lymphoma, remain significant concerns.

Postmastectomy radiation therapy (PMRT) is frequently administered to reduce the risk of breast cancer recurrence and mortality in patients who have undergone mastectomy and have axillary lymph-node metastasis [6]. Although PMRT positively impacts oncological outcomes, it can adversely affect reconstructed breasts and increase the risk of complications and reconstruction failure, particularly in implant-based reconstructions compared with autologous reconstructions [7, 8]. To clarify the safety of PMRT in implant-based breast reconstruction, this systematic review and meta-analysis was conducted in patients with breast cancer who underwent mastectomy followed by immediate implant-based breast reconstruction.

## Materials and methods

The findings of this study are reported in accordance with the Preferred Reporting Items for Systematic Reviews and Meta-Analyses (PRISMA) guidelines. This systematic review and meta-analysis were conducted as part of The Japanese Breast Cancer Society's Clinical Practice Guidelines for Radiation Treatment of Breast Cancer, 2022 edition [9].

### Eligibility criteria

Studies evaluating the effects of PMRT on immediate implant-based breast reconstruction in patients with breast cancer were included. Articles comparing two groups, irradiated and non-irradiated implant-based reconstructed breasts, were eligible for analysis. Irradiation of both the implant and expander was acceptable. Randomized or non-randomized control studies, as well as prospective or retrospective observational studies, were eligible for inclusion. The search was limited to studies written in English or Japanese that focused on human populations. Review articles, case reports with fewer than ten patients in one arm, single-arm studies, studies on delayed breast reconstruction (performed after PMRT), and studies that did not evaluate the specified outcomes were excluded.

### Search strategy

This review is an updated version of a previous systematic review and meta-analysis conducted according to the 2018 Japanese Breast Cancer Society's Clinical Practice Guidelines for Breast Cancer. The earlier systematic review and meta-analysis utilized data from PubMed/MEDLINE, the Cochrane Library, and Ichushi-Web, covering literature published from the earliest dates up to November 2016. To update this meta-analysis, a systematic literature search was performed using the same databases, PubMed/MEDLINE, the Cochrane Library, and Ichushi-Web, for studies published from January 2016 to March 2021. Literature was searched using a combination of MeSH terms and keywords, including “Breast Neoplasms,” “Radiotherapy,” “Mammaplasty,” “Autografts,” “Transplantation, Autologous,” “Breast Implants,” and “Breast Reconstruction” (Supplementary Table 1). In addition to the systematic search, relevant articles cited in the reference lists of existing review papers were manually screened. Studies included in the previous meta-analysis were re-evaluated and combined with newly selected articles for inclusion in the current analysis.

### Selection and data collection process

For the initial screening, two reviewers (SS and HJ) independently assessed the literature based on the eligibility criteria by reviewing the titles and abstracts. In the second screening, the same two reviewers (SS and HJ) independently examined the full-text articles. Any conflicts were resolved through discussion between the two reviewers or consultation with a third reviewer (MO). Data were extracted independently by two reviewers (SS and HJ) using a standardized form.

### Outcomes

The evaluated outcomes included major complications, reconstruction failure, capsular contracture, and cosmetic results. Major complications were defined as events requiring unplanned surgical intervention and/or hospitalization. Reconstruction failure was defined as the permanent removal of the expander or implant and/or conversion to autologous reconstruction. Capsular contracture was defined as cases classified as Baker grade III or IV and/or those requiring surgical intervention. Regarding cosmetic outcomes, a rating of “good” or above was considered retained cosmesis, whereas ratings below “good” were regarded as a decline in cosmesis.

### Study risk of bias and certainty assessment

Risk of bias and certainty were assessed for non-randomized studies using the Medical Information Distribution Service tool by two independent reviewers (SS and HJ) [10]. Each study was evaluated for levels of indirectness, inconsistency, imprecision, and publication bias, which led to an overall determination of the risk of bias. Subsequently, the body of evidence was established for each outcome.

### Data synthesis

A meta-analysis was conducted to compare outcomes between patients who underwent PMRT to the implant-based reconstructed breast and those who did not receive PMRT. Odds ratios (OR) and corresponding 95% confidence intervals (CI) were calculated using a random-effects models with the inverse-variance method. Publication bias was assessed using funnel plots. All statistical tests were two-sided, and *P* values < 0.05 were considered statistically significant. Statistical analysis was performed using Review Manager (RevMan) v5.4 software developed by the Cochrane Collaboration.

## Results

### Study selection and characteristics

**Fig. 1:**
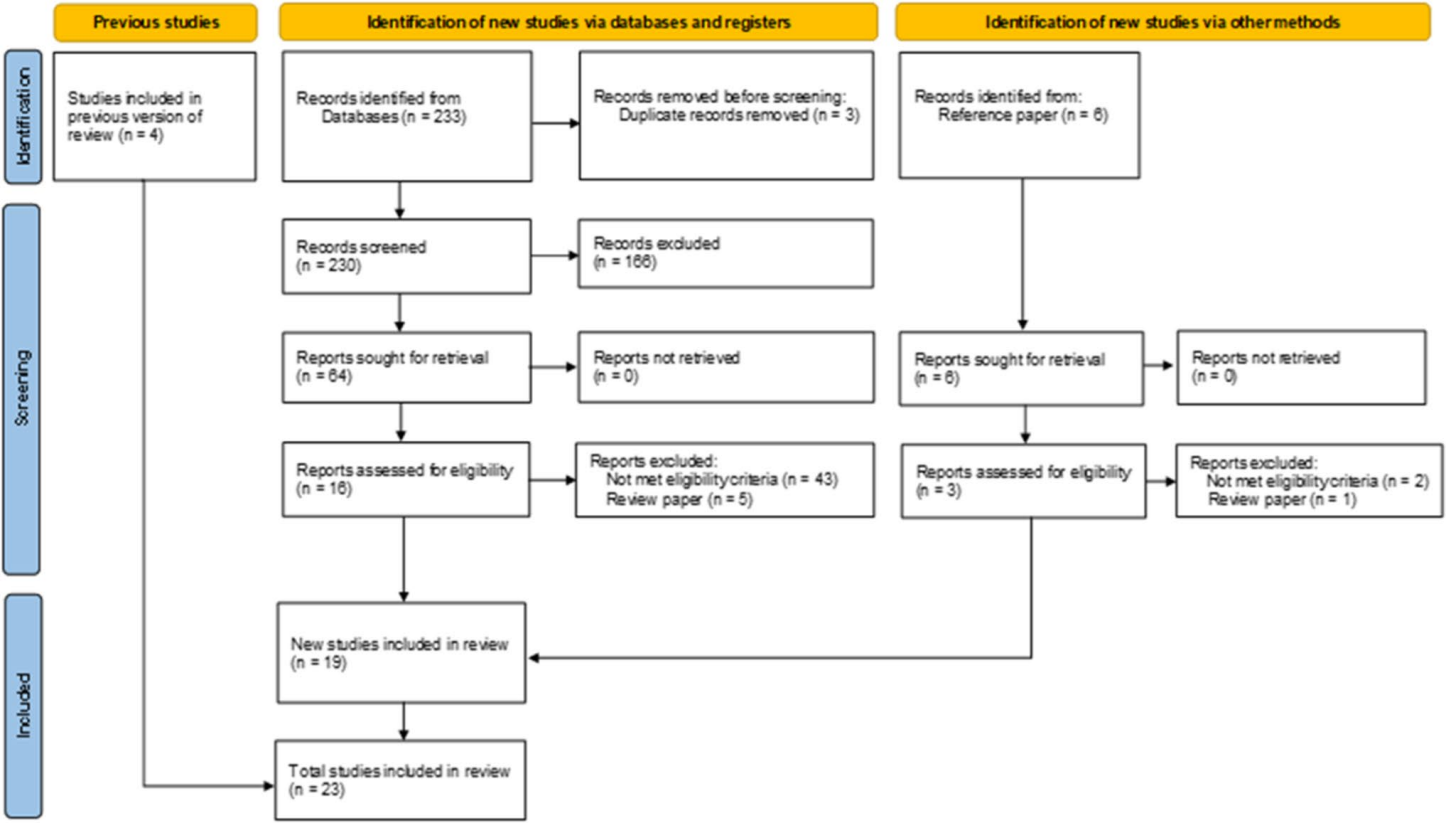
PRISMA 2020 flow diagram of the literature screening process

A total of 233 citations were identified through the systematic search. After removing three duplicates, 230 citations remained. Following the initial screening, 166 citations were excluded, and 64 studies were subjected to full-text screening. Of these, 48 were excluded, leaving 19 studies that met the inclusion criteria. Including four studies from previous analyses, a total of 23 studies, comprising one case-controlled study, one prospective cohort study, and 21 retrospective cohort studies, were included in this meta-analysis (Fig. 1). The characteristics of these studies are presented in Table 1.

**Table 1:** Characteristics of the included studies in the meta-analysis

Study	Institution	Study period	Study design	Number of patients or breasts for analysis	Follow-up period (months)	Type of reconstruction	RT dose to the reconstructed breast	Boost	Bolus
Behranwal, K.A. 2006	Royal Marsden NHS Foundation Trust, UK	1998–2001	Retrospective Cohort	Total 136, RT 44, No RT 92	Median 48 (range, 24–60)	One stage	Median 50.4 Gy (range, 50–60)	NA	NA
Patani, N. 2008	Three Independent Sector Healthcare Centres in London, UK	2001–2007	Retrospective Cohort	Total 83, RT 16, No RT 67	Median 34 (range, 3–79)	Two stage	NA	NA	NA
Whitfield, G.A. 2008	Cambridge University Hospitals NHS Trust, UK	2001–2005	Retrospective Cohort	Total 120, RT 42, No RT 78	Median 51	One/two stage	40 Gy/15 fr	NA	Yes
Drucker-Zertuche, M. 2011	National Cancer Institute of Mexico City, Mexico	2002–2008	Retrospective Cohort	Total 97, RT 37, No RT 60	Mean 39.2 (range, 4–72), RT 40.2 (4–72), No RT 38.4 (4–68)	Two stage	50 Gy/25 fr	NA	NA
Jimenez-Puente, A. 2011	Costa del Sol Hospital, Spain	2002–2009	Retrospective Cohort	Total 115, RT 32, No RT 83	Mean 25.5 (range, 9–78)	Two stage	45–50.4 Gy, 1.8–2.0 Gy/fr	NA	NA
Lin, K.Y. 2011	University of Virginia School of Medicine, US	2000–2009	Retrospective Cohort	Total 235, RT 17, No RT 218	Mean RT 28.4, No RT 26.3	Two stage	50 Gy/25 fr	NA	NA
Nava, M.B. 2011	Fondazione IRCCS Istituto Nazionale dei Tumori, Italy	2003–2007	Case–Control	Total 257, RT 159, No RT 98	Median 50	Two stage	51 Gy/25–28 fr	NA	NA
Cordeiro, P.G. 2014	Memorial Sloan-Kettering Cancer Center, US	1998–2010	Retrospective Cohort	Total 2133, RT 319, No RT 1814	Mean 56.8 (range, 12–164), RT 54.4 (12–164), No RT 57.3 (12–164)	Two stage	NA	NA	Yes
Anker, C.J. 2015	CancerCare Manitoba, Canada	2012–2015	Retrospective Cohort	Total 222, RT 61, No RT 161	Mean 44 (range, 6–144)	Two stage	Median 50.4 Gy/28fr	Scar boost 10 Gy (41% of irradiated patients)	Yes (59% of irradiated patients)
Kearney, A.M. 2015	Case Medical Center, US	2007–2013	Retrospective Cohort	Total 242, RT 33, No RT 209	Mean 19.6 (range, 3–69)	Two stage	NA	NA	NA
Chen, T.A. 2016	Stanford University, US	2007–2013	Retrospective Cohort	Total 68, RT 38, No RT 30	NA	Two stage	NA	NA	NA
Matsukata, A. 2016	Sagara Hospital, Japan	2007–2014	Retrospective Cohort	Total 100, RT 27, No RT 73	Median RT 37 (15–99), No RT 34 (12–99)	Two stage	50 Gy/25 fr	NA	No
Chuba, P.J. 2017	The St John Hospital and Medical Center and the St John Macomb Oakland Hospital, US	2003–2013	Retrospective Cohort	Total 212, RT 127, No RT 85	NA	One/two stage (mostly two stage)	50 Gy, 1.8–2.0 Gy/fr	NA	Yes
Pompei, S. 2017	Sandro Pertini Hospital, Italy	2002–2015	Retrospective Cohort	Total 115, RT 56, No RT 59	Median 103.3 (range, 6–152), RT 106.6 (6–152), No RT 103.9 (34–152)	Two stage	Mean 50 Gy	NA	NA
Elswick, S.M. 2018	Mayo Clinic, US	2012–2016	Retrospective Cohort	Total 93, RT 54 No RT 39	Mean 19 (range, 1–36)	Two stage	Median 50 Gy/25fr (range, 49–60 Gy/25–30 fr)	NA	NA
Lam, T.C. 2018	Westmead Public Hospital, Australia	1998–2010	Retrospective Cohort	Total 452, RT 114, No RT 338	Mean RT 39.6, No RT 44.9	Two stage	50 Gy/25 fr	NA	No
Sinnott, C.J. 2018	South Nassau Communities Hospital and Yale New Haven Health Bridgeport Hospital, US	2010–2017	Retrospective Cohort	Total 589, RT 79, No RT 510	NA	One/two stage	50 Gy/25 fr	NA	NA
Riggio, E. 2019	Fondazione IRCCS Istituto Nazionale dei Tumori, Italy	2011–2014	Retrospective Cohort	Total 224, RT 44, No RT 180	Median 26.3	One/two stage	NA	NA	NA
Hamann, M. 2019	Red Cross Hospital Munich, Germany	2008–2012	Retrospective Cohort	Total 89, RT 26, No RT 63	Median 17.8, RT 16.6 (3–50), No RT 17.0 (4–61)	Two stage	50	NA	NA
Zhang, L. 2019	Fudan University Shanghai Cancer Center, China	2001–2015	Retrospective Cohort	Total 394, RT 52, No RT 342	Median 58.5 (range, 12–176)	One/two stage	NA	NA	NA
Naoum, G.E. 2020	Massachusetts General Hospital, US	1997–2017	Retrospective Cohort	Total 839, RT 236, No RT 603	Median 69.6, RT 75, No RT 80.0	Two stage	Median 50.4 Gy (range, 45–68)	Yes (49.6%)	Yes
Sewart, E. 2020	81 UK Breast and Plastic Surgical Units, UK	2014–2016	Prospective Cohort	Total 730, RT 214, No RT 516	NA	One/two stage	NA	NA	NA
Chen, J.J. 2021	Stanford University School of Medicine, US	2000–2014	Retrospective Cohort	Total 68, RT 38, No RT 30	Median 42.5 (range, 1–164)	Two stage	Median 50.4 Gy (range, 37.5–61)	NA	NA

RT, radiation therapy; NA, not applicable

### Risk-of-bias assessment

Risk of bias, indirectness, and inconsistency for each outcome in the included studies are detailed in Supplementary Tables 2, 3, 4, and 5. The studies included in this analysis were observational and neither blinded nor randomized. Only one study adjusted for confounding factors, indicating a risk of bias across all evaluated outcomes. Although variations were present in irradiation methods, fields, and outcome definitions across studies, no significant indirectness was observed for any of the outcomes. Inconsistency was low for capsular contracture and moderate for the other outcomes. Publication bias was detected for major complications (Supplementary Fig. S1, S2, S3, and S4).

### Results of synthesis

#### Major complications

**Fig. 2:**
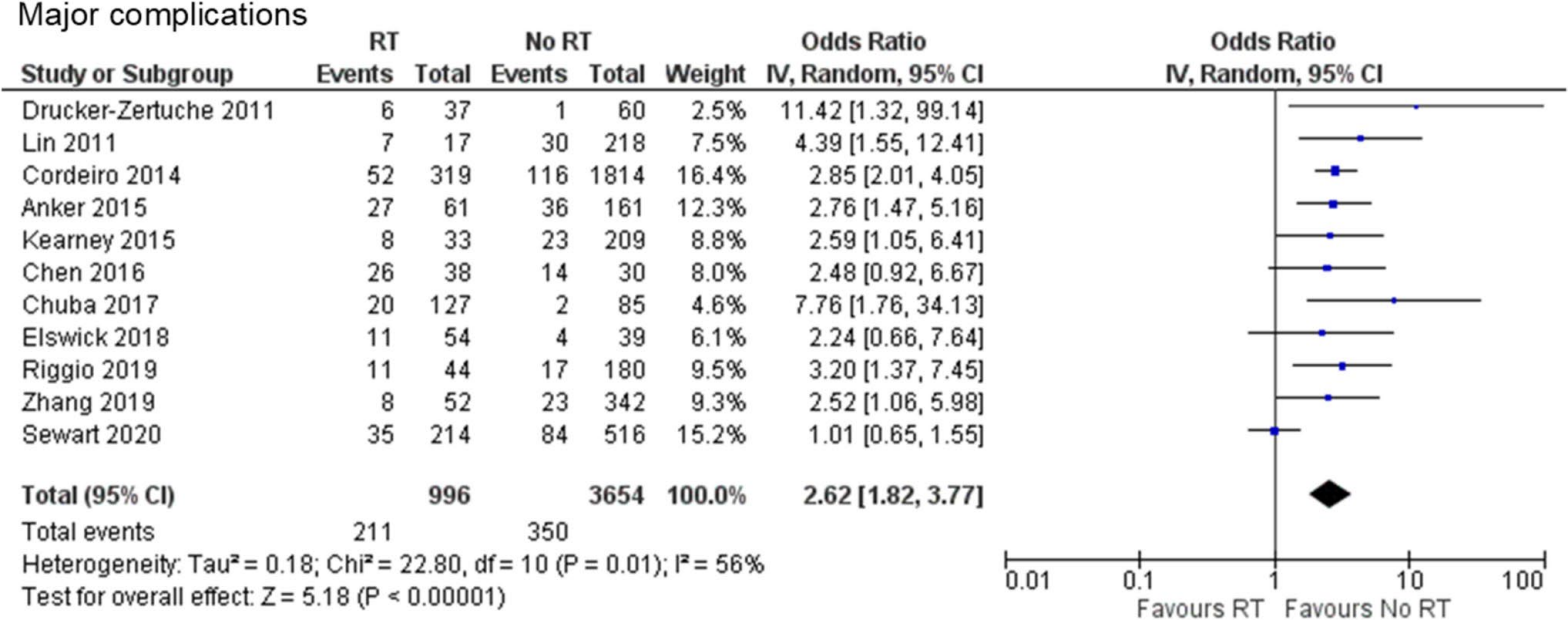
Forest plot illustrating the impact of PMRT on major complications. PMRT, postmastectomy radiation therapy; IV, inverse variance

Eleven retrospective cohort studies were included in the analysis of major complications [11–21]. A total of 4,650 cases were analyzed, including 996 and 3,654 cases in the PMRT and no PMRT groups, respectively. The incidence of major complications was significantly higher in the PMRT group than in the no PMRT group (OR 2.62, 95% CI 1.82–3.77, *P* < 0.00001), with substantial heterogeneity (Chi^2^ = 22.80, df = 10, *P* = 0.01, I2 = 56%) (Fig. 2).

#### Reconstruction failure

**Fig. 3:**
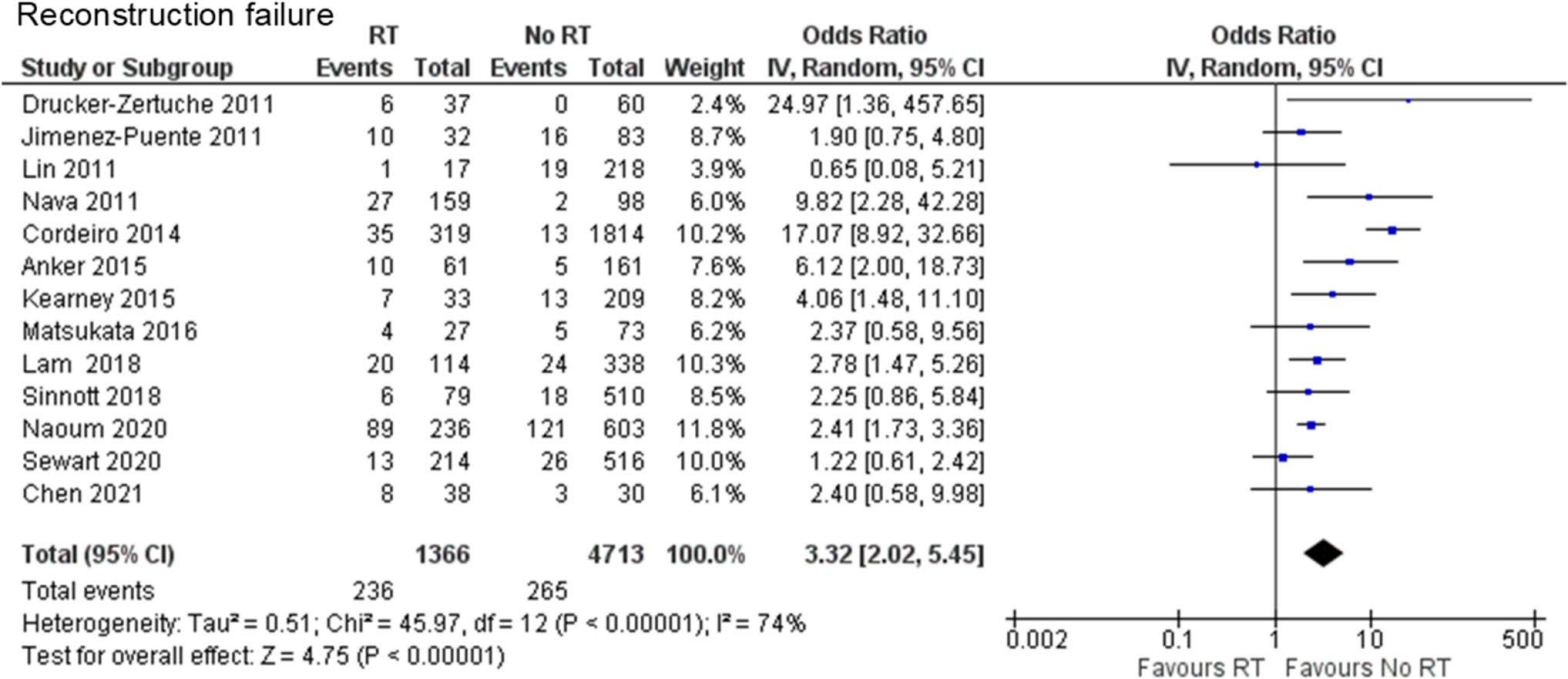
Forest plot illustrating the impact of PMRT on reconstruction failure. PMRT, postmastectomy radiation therapy; IV, inverse variance

One case–control study [22] and 12 retrospective cohort studies [11, 12, 16, 17, 19, 20, 23–28] were included in the analysis of reconstruction failure. A total of 6,079 cases were analyzed: 1,366 in the PMRT group and 4,713 in the no PMRT group. Reconstruction failure was significantly more frequent in the PMRT group than in the no PMRT group (OR 3.32, 95% CI 2.02–5.45, *P* < 0.00001), with substantial heterogeneity (Chi^2^ = 45.97, df = 12, *P* < 0.00001, I^2^ = 74%) (Fig. 3).

#### Capsular contracture

**Fig. 4:**
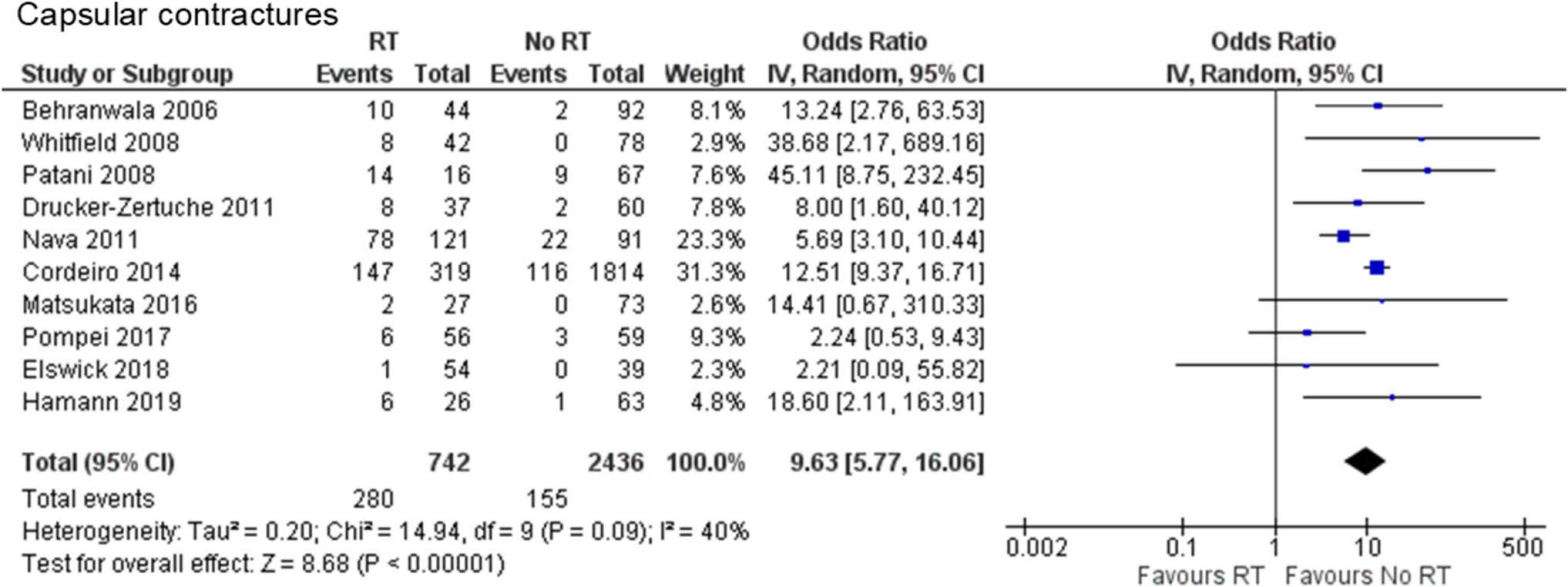
Forest plot illustrating the impact of PMRT on capsular contracture. PMRT, postmastectomy radiation therapy; IV, inverse variance

One case–control study [22] and nine retrospective cohort studies [14, 17, 24, 29–33] were included to evaluate capsular contracture. A total of 3,178 cases were analyzed: 742 in the PMRT group and 2,436 in the no PMRT group. Capsular contracture with Baker grade III or IV and/or requiring surgical intervention occurred significantly more frequently in the PMRT group than in the no PMRT group (OR 9.63, 95% CI 5.77–16.06, P < 0.00001), with no substantial heterogeneity (Chi^2^ = 14.94, df = 9, *P* = 0.09, I^2^ = 40%) (Fig. 4).

#### Cosmesis

**Fig. 5:**
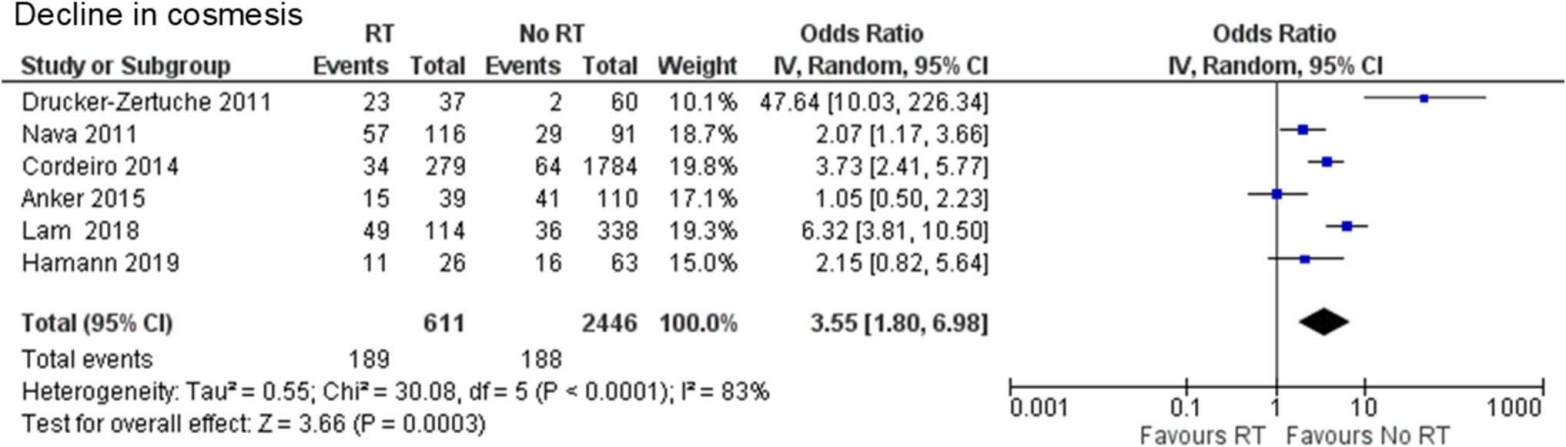
Forest plot illustrating the impact of PMRT on cosmesis. PMRT: postmastectomy radiation therapy; IV: inverse variance

One case–control study [22] and five retrospective cohort studies [16, 17, 20, 28, 33] were included in the assessment of cosmesis. A total of 3,057 cases were analyzed: 611 in the PMRT group and 2,446 in the no PMRT group. A decline in cosmesis was observed significantly more often in the PMRT group than in the no PMRT group (OR 3.55, 95% CI 1.80–6.98, P = 0.0003), with substantial heterogeneity (Chi^2^ = 30.08, df = 5, *P* < 0.0001, I^2^ = 83%) (Fig. 5).

#### Certainty of evidence

Supplementary Table S6 summarizes the overall body of evidence for each outcome. The certainty of evidence was rated as low across all outcomes, primarily due to study limitations and potential publication bias.

## Discussion

This systematic review and meta-analysis evaluated the impact of PMRT on implant-based breast reconstruction in patients with breast cancer. The findings indicate that PMRT is significantly associated with an increased risk of adverse events, including major complications, reconstruction failure, capsular contracture, and declining aesthetic outcomes, in patients undergoing implant-based breast reconstruction. As the indication for PMRT is primarily determined by breast cancer risks, and oncologic outcomes must take precedence, its use is often unavoidable, even in patients undergoing implant-based reconstruction. Therefore, before initiating treatment, physicians should engage in detailed discussions with patients regarding the available reconstructive options and their associated risks and benefits.

The results indicated that patients receiving PMRT had approximately twofold and threefold increased odds of experiencing major complications and reconstruction failure, respectively, compared with those not receiving PMRT. Capsular contracture showed a particular increase, with nearly tenfold higher odds, and cosmetic outcomes significantly declined in the PMRT group. These findings are consistent with those of previous studies.

Several meta-analyses have evaluated the effects of PMRT on implant-based reconstruction and consistently reported an increased risk of complications; however, each study had notable limitations. Barry et al. (2011) reported that PMRT increased the overall risk of postoperative complications, which encompassed various issues, including infection and capsular contracture, although the study was limited by a very small sample size [7]. Similarly, Lam et al. (2013) examined reconstruction failure and capsular contracture and found higher rates of both in the PMRT group; however, their analysis was limited by the small number of studies and patients [34]. Magill et al. (2017) expanded upon these earlier findings by evaluating a broader set of outcomes, including capsular contracture, reconstruction failure, revision surgery, cosmetic results, and patient satisfaction. Their analysis offered a more comprehensive perspective, demonstrating that PMRT was significantly associated with increased rates of reconstructive failure, capsular contracture, and revision surgery, as well as decreased cosmetic outcomes and patient satisfaction [35]. However, despite including a large sample size than previous analyses, the overall cohort size remained modest. The most recent meta-analysis by Pu et al. (2018) included a comparatively larger sample and assessed multiple outcomes, including overall complications, reconstruction failure, capsular contracture, and patient satisfaction. The analysis revealed significant increases in overall complications, reconstruction failure, and capsular contracture following PMRT [36]. Nevertheless, despite its broader scope, this review did not systematically assess the quality of the included studies.

The present meta-analysis represents the comprehensive and methodologically robust evaluation to date. It includes the largest number of studies and patients in this context and incorporates the most recent literature. Balanced and clinically relevant outcomes, including major complications, reconstruction failure, capsular contracture, and aesthetic results, were systematically evaluated. Additionally, the quality of each included study was assessed, including the risk of bias, indirectness, and inconsistency. This assessment provides a crucial level of interpretive certainty that was lacking in previous studies.

The timing of PMRT is also an important clinical factor that may influence reconstructive outcomes. The effects of PMRT delivered during tissue expander placement versus after implant exchange may vary in terms of complication rates. While this study focused on the overall outcomes of PMRT in implant-based reconstruction, we have conducted a separate meta-analysis examining PMRT timing, which has been accepted for publication and is currently in press.

Although implant-based reconstruction remains a widely used approach for postmastectomy breast reconstruction, the present findings highlight that outcomes may be negatively affected by PMRT. Therefore, careful patient selection and individualized pretreatment counseling are essential when considering breast reconstruction and PMRT. Clinicians should also consider alternative reconstructive strategies for patients likely to undergo PMRT, particularly autologous tissue reconstruction, which has been associated with more favorable outcomes. A prospective multicenter cohort study demonstrated that autologous reconstruction was associated with a lower risk of complications compared with implant-based reconstruction at 2 years and higher BREAST-Q satisfaction scores among patients who received PMRT [8]. Similarly, a meta-analysis by Ren et al. comparing immediate autologous and immediate implant-based reconstruction in the setting of PMRT reported higher reconstruction failure rates in the implant-based group, although the rates of complications requiring reoperation were comparable between the two methods [37]. The Oncoplastic Breast Consortium recommends autologous breast reconstruction as the preferred reconstruction method over implants in patients requiring radiation therapy [38].

This study has several limitations. Most of the included studies were retrospective, which may have introduced selection and reporting biases. Furthermore, variations in patient populations, surgical techniques, radiation modalities, and follow-up durations across studies may have contributed to the heterogeneity observed in the pooled analysis. The assessment of aesthetic outcomes was often subjective and lacked standardized evaluation systems, which limits comparability. The reconstructive plane (prepectoral vs. subpectoral) can affect PMRT-related risks and aesthetic outcomes. However, most cohorts in the included studies were subpectoral, and prepectoral cohorts were comparatively few. Accordingly, plane-stratified meta-analysis was not feasible, and our pooled estimates are most applicable to subpectoral reconstruction. Future studies should report outcomes separately by plane and include key technical details, such as acellular dermal matrix use, to enable robust plane-specific analyses. Despite these limitations, the consistency in the study results and outcomes provides compelling evidence of the risks associated with PMRT in implant-based breast reconstruction.

In conclusion, this meta-analysis demonstrated that PMRT has a significantly negative impact on implant-based breast reconstruction. Individualized treatment and patient counseling are essential when considering reconstruction in the context of PMRT. A multidisciplinary collaboration between surgeons and radiation oncologists is necessary to optimize both oncological and reconstructive safety.

## Supplementary Information

Below is the link to the electronic supplementary material.

Supplementary file1 (DOCX 112 KB)

## Data Availability

All data used in this meta-analysis were extracted from publicly available articles cited in the manuscript. The studylevel extraction dataset compiled for this review is available from the corresponding author upon reasonable request.
