# Supplementary material for: Postmastectomy radiation therapy for implant-based breast reconstruction: a systematic review and meta-analysis for the 2022 Japanese Breast Cancer Society Clinical Practice Guideline
Source: Breast Cancer. 2025 Sep 30;32(6):1169–79. doi: 10.1007/s12282-025-01788-2 (PMC12552255; doi:10.1007/s12282-025-01788-2)
Supplement: Supplementary file 1 — Supplementary file1 (DOCX 112 KB) [file 12282_2025_1788_MOESM1_ESM.docx]

**Supplementary Appendix**

Supplementary Table 1. Search strategies on PubMed/MEDLINE

| No | Search Strategy | Result |
| --- | --- | --- |
| #01 | "Breast Neoplasms/radiotherapy"[Mesh] OR ("Breast Neoplasms/therapy"[Mesh] AND "Radiotherapy"[Mesh]) | 20,211 |
| #02 | "Mammaplasty"[Mesh] | 14,682 |
| #03 | "Autografts"[Mesh] OR "Transplantation, Autologous"[Mesh] OR "Breast Implants"[Mesh] | 58,869 |
| #04 | #1 AND #2 AND #3 | 260 |
| #05 | (Breast[TI] OR Mammary[TI]) AND (Tumo*[TI] OR Cancer*[TIAB] OR Carcinoma*[TIAB] OR Neoplasm*[TIAB] OR oncology[TIAB]) AND (mammaplast*[TIAB] OR mammoplast*[TIAB] OR "breast reconstruction"[TIAB]) AND (Radiotherapy[TIAB] OR "radiation therapy"[TIAB] OR irradiation[TIAB]) AND (Autograft*[TIAB] OR Autologous[TIAB] OR implant*[TIAB]) | 386 |
| #06 | #4 OR #5 | 576 |
| #07 | #6 AND 2016/1:2021/3[DP] | 252 |
| #08 | #7 AND (JAPANESE[LA] OR ENGLISH[LA]) | 248 |
| #09 | #8 AND ("Meta-Analysis"[PT] OR "Meta-Analysis as Topic"[Mesh] OR "meta-analysis"[TIAB]) | 14 |
| #10 | #8 AND ("Cochrane Database Syst Rev"[TA] OR "Systematic Review"[PT] OR "Systematic Reviews as Topic"[Mesh] OR "systematic review"[TIAB]) | 13 |
| #11 | #8 AND ("Practice Guideline"[PT] OR "Practice Guidelines as Topic"[Mesh] OR "Consensus"[Mesh] OR "Consensus Development Conferences as Topic"[Mesh] OR "Consensus Development Conference"[PT] OR guideline*[TI] OR consensus[TI]) | 3 |
| #12 | #9 OR #10 OR #11 | 20 |
| #13 | #8 AND ("Randomized Controlled Trial"[PT] OR "Randomized Controlled Trials as Topic"[Mesh] OR (random*[TIAB] NOT medline[SB])) | 6 |
| #14 | #8 AND ("Clinical Trial"[PT] OR "Clinical Trials as Topic"[Mesh] OR "Observational Study"[PT] OR "Observational Studies as Topic"[Mesh] OR (("clinical trial"[TIAB] OR "case control"[TIAB] OR "case comparison"[TIAB]) NOT medline[SB])) | 15 |
| #15 | (#13 OR #14) NOT #12 | 17 |
| #16 | #8 AND ("Epidemiologic Methods"[Mesh] OR "Comparative Study"[PT] OR "Multicenter Study"[PT] OR "Validation Study"[PT] OR ((cohort*[TIAB] OR "comparative study"[TIAB] OR "follow-up"[TIAB] OR "prospective study"[TIAB] OR "Retrospective study"[TIAB]) NOT medline[SB])) | 167 |
| #17 | #16 NOT (#12 OR #15) | 147 |

Supplementary Table 2. Risk of bias assessment for major complications

| Outcomes | | Major complications requiring a surgical intervention and/or hospitalization | | | | | | | | | |  |  | |  | | | |  | |  |  | |  | | |  | |  | | |  |  |  |  |  |  |  |  |  |  |
| --- | --- | --- | --- | --- | --- | --- | --- | --- | --- | --- | --- | --- | --- | --- | --- | --- | --- | --- | --- | --- | --- | --- | --- | --- | --- | --- | --- | --- | --- | --- | --- | --- | --- | --- | --- | --- | --- | --- | --- | --- | --- |
| Individual study | | Risk of bias | | | | | | | | | |  |  | |  | | | |  | |  |  | |  | | |  | |  | | |  |  |  |  |  |  |  |  |  |  |
|  |  | Selection bias | | Performance bias | | Detection bias | Attrition bias | | Others | | |  | Factors that can increase the certainty of the evidence | | | | | | | |  | Indirectness | | | | | | | | | |  | Number at risk (outcome rate) | | | | | |  |  |  |
| Study code | Study design | Differences between baseline characteristics | | | Differences between groups in the care | Differences between groups in how outcomes are determined | Differences between groups in withdrawals | | Inadequate adjustment for confounding | Others | | Summary | | Dose-response gradient | | Effect of potential residual confounding factors | | Large magnitude of effect | | | Summary | Participants | | | Intervention | | | Comparison | | Outcome | | Summary | The denominator for comparison arm | The numerator for comparison arm | (%) | The denominator for intervention arm | The numerator for intervention arm | (%) | Effect measures | An estimate of effect | 95% confidence interval |
| Lin, K.Y. 2011 | Retrospective cohort | -2 | | | -1 | -2 | 0 | | -2 | 0 | | -1 | | 0 | | 0 | | 0 | | | 0 | 0 | | | 0 | | | 0 | | 0 | | 0 | 60 | 1 | 1.7 | 37 | 6 | 16.2 | OR | 11.42 | 1.32–99.14 |
| Sewart, E. 2020 | Prospective cohort | -2 | | | -2 | -2 | 0 | | -2 | 0 | | -2 | | 0 | | 0 | | 0 | | | 0 | 0 | | | 0 | | | 0 | | 0 | | 0 | 218 | 30 | 13.8 | 17 | 7 | 41.2 | OR | 4.39 | 1.55–12.41 |
| Zhang, L. 2019 | Retrospective cohort | -2 | | | -1 | -2 | 0 | | -2 | 0 | | -1 | | 0 | | 0 | | 0 | | | 0 | -1 | | | 0 | | | 0 | | 0 | | 0 | 1814 | 116 | 6.4 | 319 | 52 | 16.3 | OR | 2.85 | 2.01–4.05 |
| Elswick, S.M. 2018 | Retrospective cohort | -2 | | | -2 | -2 | 0 | | -2 | 0 | | -2 | | 0 | | 0 | | 0 | | | 0 | 0 | | | 0 | | | 0 | | 0 | | 0 | 161 | 36 | 22.4 | 61 | 27 | 44.3 | OR | 2.76 | 1.47–5.16 |
| Chuba, P.J. 2017 | Retrospective cohort | -2 | | | -2 | -2 | 0 | | -2 | 0 | | -2 | | 0 | | 0 | | 0 | | | 0 | 0 | | | 0 | | | 0 | | 0 | | 0 | 209 | 23 | 11.0 | 33 | 8 | 24.2 | OR | 2.59 | 1.05–6.41 |
| Cordeiro, P.G. 2014 | Retrospective cohort | -2 | | | 0 | -2 | 0 | | -2 | 0 | | -1 | | 0 | | 0 | | 0 | | | 0 | 0 | | | 0 | | | 0 | | -2 | | -1 | 30 | 14 | 46.7 | 38 | 26 | 68.4 | OR | 2.48 | 0.92–6.67 |
| Drucker-Zertuche, M. 2011 | Retrospective cohort | -2 | | | -1 | -2 | 0 | | -2 | 0 | | -1 | | 0 | | 0 | | 0 | | | 0 | -1 | | | 0 | | | 0 | | 0 | | 0 | 85 | 2 | 2.4 | 127 | 20 | 15.7 | OR | 7.76 | 1.76–34.13 |
| Chen, T.A. 2016 | Retrospective cohort | -2 | | | -1 | -2 | 0 | | -2 | 0 | | -1 | | 0 | | 0 | | 0 | | | 0 | 0 | | | 0 | | | 0 | | 0 | | 0 | 39 | 4 | 10.3 | 54 | 11 | 20.4 | OR | 2.24 | 0.66–7.64 |
| Kearney, A.M. 2015 | Retrospective cohort | -2 | | | -1 | -2 | 0 | | -2 | 0 | | -1 | | 0 | | 0 | | 0 | | | 0 | 0 | | | 0 | | | 0 | | 0 | | 0 | 180 | 17 | 9.4 | 44 | 11 | 25.0 | OR | 3.2 | 1.37–7.45 |
| Anker, C.J. 2015 | Retrospective cohort | -2 | | | -2 | -2 | 0 | | -2 | 0 | | -2 | | 0 | | 0 | | 0 | | | 0 | -2 | | | 0 | | | 0 | | -2 | | -1 | 342 | 23 | 6.7 | 52 | 8 | 15.4 | OR | 2.52 | 1.06–5.98 |
| Riggio, E. 2019 | Retrospective cohort | -2 | | | -2 | -2 | -2 | | -2 | 0 | | -2 | | 0 | | 0 | | 0 | | | 0 | 0 | | | 0 | | | 0 | | -2 | | -1 | 516 | 84 | 16.3 | 214 | 35 | 16.4 | OR | 1.01 | 0.65–1.55 |
| Comment |  | |  | | |  | |  | | |  | |  | | | |  | | |  | | |  | | |  | | | | |  | |  |  |  |  |  |  |  |  |  |
| Drucker-Zertuche, M. 2011 |  | Differences in TNM stage were expected. | | | Difference in chemotherapy and endocrine therapy were expected. | Unblinded |  | | No adjustment for confounding was performed. |  | |  | |  | |  | |  | | |  |  | | |  | | |  | |  | |  |  |  |  |  |  |  |  |  |  |
| Lin, K.Y. 2011 |  | TNM status unknown. | | | Difference in chemotherapy. | Unblinded |  | | No TNM adjustment was performed in the multivariate analysis. |  | |  | |  | |  | |  | | |  |  | | |  | | |  | |  | |  |  |  |  |  |  |  |  |  |  |
| Cordeiro, P.G. 2014 |  | Differences in TNM stage were expected. | | | Difference in chemotherapy and endocrine therapy were expected. | Unblinded |  | | No adjustment for confounding was performed. |  | |  | |  | |  | |  | | |  |  | | |  | | |  | |  | |  |  |  |  |  |  |  |  |  |  |
| Anker, C.J. 2015 |  | Difference in TNM stage. | | | Difference in chemotherapy. | Unblinded |  | | No adjustment for confounding was performed. |  | |  | |  | |  | |  | | |  |  | | |  | | |  | |  | |  |  |  |  |  |  |  |  |  |  |
| Kearney, A.M. 2015 |  | Differences in TNM stage were expected. | | | Difference in chemotherapy. | Unblinded |  | | No adjustment for confounding was performed. |  | |  | |  | |  | |  | | |  |  | | |  | | |  | |  | |  |  |  |  |  |  |  |  |  |  |
| Chen, T.A. 2016 |  | Differences in TNM stage were expected. | | |  | Unblinded |  | | No adjustment for confounding was performed. |  | |  | |  | |  | |  | | |  |  | | |  | | |  | | No definition of major complication | |  |  |  |  |  |  |  |  |  |  |
| Chuba, P.J. 2017 |  | Differences in TNM stage were expected. | | | Difference in chemotherapy and endocrine therapy were expected. | Unblinded |  | | No adjustment for confounding was performed. |  | |  | |  | |  | |  | | |  | Irradiation group contained 5% non-PMRT. | | |  | | |  | |  | |  |  |  |  |  |  |  |  |  |  |
| Elswick, S.M. 2018 |  | Differences in TNM stage were expected. | | | Difference in chemotherapy and endocrine therapy were expected. | Unblinded |  | | No adjustment for confounding was performed. |  | |  | |  | |  | |  | | |  |  | | |  | | |  | |  | |  |  |  |  |  |  |  |  |  |  |
| Riggio, E. 2019 |  | Differences in TNM stage were expected. | | | Difference in chemotherapy and endocrine therapy were expected. | Unblinded |  | | No adjustment for confounding was performed. |  | |  | |  | |  | |  | | |  |  | | |  | | |  | |  | |  |  |  |  |  |  |  |  |  |  |
| Zhang, L. 2019 |  | TNM status unknown. | | | Difference in chemotherapy. | Unblinded |  | | No TNM adjustment was performed in the multivariate analysis. |  | |  | |  | |  | |  | | |  | Some delayed reconstruction was included. | | |  | | |  | | Reoperation inicluded balancing operations. | |  |  |  |  |  |  |  |  |  |  |
| Sewart, E. 2020 |  | Large size in PMRT group | | | More patients received chemotherapy in PMRT group. | Unblinded | Short follow-up duration (3 months) | | No correction in multivariate analysis. |  | |  | |  | |  | |  | | |  |  | | |  | | |  | | Results within 3 months only | |  |  |  |  |  |  |  |  |  |  |

Supplementary Table 3. Risk of bias assessment for reconstruction failure

| Outcomes | | | Reconstruction failure | | | | | | | | | |  | |  | |  | |  | | |  |  | |  | |  | | |  | |  | |  |  | |  | |  | |  | |  |  | |  | | | |  |
| --- | --- | --- | --- | --- | --- | --- | --- | --- | --- | --- | --- | --- | --- | --- | --- | --- | --- | --- | --- | --- | --- | --- | --- | --- | --- | --- | --- | --- | --- | --- | --- | --- | --- | --- | --- | --- | --- | --- | --- | --- | --- | --- | --- | --- | --- | --- | --- | --- | --- | --- |
| Individual study | | | Risk of bias | | | | | | | | | |  | |  | |  | |  | | |  |  | |  | |  | | |  | |  | |  |  | |  | |  | |  | |  |  | |  | | | |  |
|  |  |  | Selection bias | Performance bias | | | Detection bias | Attrition bias | | Others | | |  | | Factors that can increase the certainty of the evidence | | | | | | |  | Indirectness | | | | | | | | | |  | Number at risk (outcome rate) | | | | | | | | | | |  | | |  | | |
| Study code | Study design | | Differences between baseline characteristics | Differences between groups in the care | | Differences between groups in how outcomes are determined | | Differences between groups in withdrawals | | Inadequate adjustment for confounding | | Others | Summary | | | Dose-response gradient | | Effect of potential residual confounding factors | | Large magnitude of effect | | Summary | Participants | | | Intervention | | | Comparison | | Outcome | | Summary | The denominator for comparison arm | | The numerator for comparison arm | | (%) | | The denominator for intervention arm | | The numerator for intervention arm | | (%) | Effect measures | | An estimate of effect | | 95% Confidence interval | |
| Drucker-Zertuche, M. 2011 | Retrospective cohort | | -2 | -1 | | -2 | | 0 | | -2 | | 0 | -1 | | | 0 | | 0 | | 0 | | 0 | 0 | | | 0 | | | 0 | | 0 | | 0 | 60 | | 0 | | 0.0 | | 37 | | 6 | | 16.2 | OR | | 24.97 | | 1.36–457.65 | |
| Jimenez-Puente, A. 2011 | Retrospective cohort | | -2 | -1 | | -2 | | 0 | | -2 | | 0 | -2 | | | 0 | | 0 | | 0 | | 0 | 0 | | | 0 | | | 0 | | 0 | | 0 | 83 | | 16 | | 19.3 | | 32 | | 10 | | 31.3 | OR | | 1.9 | | 0.75–4.80 | |
| Lin KY, 2011 | Retrospective cohort | | -2 | -2 | | -2 | | 0 | | -2 | | 0 | -2 | | | 0 | | 0 | | 0 | | 0 | 0 | | | 0 | | | 0 | | 0 | | 0 | 218 | | 19 | | 8.7 | | 17 | | 1 | | 5.9 | OR | | 0.65 | | 0.08–5.21 | |
| Nava, M.B. 2011 | Case-control | | -2 | -1 | | -2 | | 0 | | -1 | | 0 | -1 | | | 0 | | 0 | | 0 | | 0 | 0 | | | 0 | | | 0 | | 0 | | 0 | 98 | | 2 | | 2.0 | | 159 | | 27 | | 17.0 | OR | | 9.82 | | 2.28–42.28 | |
| Cordeiro, P.G. 2014 | Retrospective cohort | | -2 | -1 | | -2 | | 0 | | -2 | | 0 | -1 | | | 0 | | 0 | | 0 | | 0 | -1 | | | 0 | | | 0 | | 0 | | 0 | 1814 | | 13 | | 0.7 | | 319 | | 35 | | 11.0 | OR | | 17.07 | | 8.92–32.66 | |
| Anker, C.J. 2015 | Retrospective cohort | | -2 | -2 | | -2 | | 0 | | -2 | | 0 | -2 | | | 0 | | 0 | | 0 | | 0 | 0 | | | 0 | | | 0 | | 0 | | 0 | 161 | | 5 | | 3.1 | | 61 | | 10 | | 16.4 | OR | | 6.12 | | 2.00–18.73 | |
| Kearney, A.M. 2015 | Retrospective cohort | | -2 | -2 | | -2 | | 0 | | -2 | | 0 | -2 | | | 0 | | 0 | | 0 | | 0 | 0 | | | 0 | | | 0 | | 0 | | 0 | 209 | | 13 | | 6.2 | | 33 | | 7 | | 21.2 | OR | | 4.06 | | 1.48–11.10 | |
| Matsukata, A. 2016 | Retrospective cohort | | -2 | -2 | | -2 | | 0 | | -2 | | 0 | -2 | | | 0 | | 0 | | 0 | | 0 | 0 | | | 0 | | | 0 | | -1 | | 0 | 73 | | 5 | | 6.8 | | 27 | | 4 | | 14.8 | OR | | 2.37 | | 0.58–9.56 | |
| Lam, T.C. 2018 | Retrospective cohort | | -2 | -2 | | -2 | | 0 | | -2 | | 0 | -2 | | | 0 | | 0 | | 0 | | 0 | 0 | | | 0 | | | 0 | | 0 | | 0 | 338 | | 24 | | 7.1 | | 114 | | 20 | | 17.5 | OR | | 2.78 | | 1.47–5.26 | |
| Sinnott, C.J. 2018 | Retrospective cohort | | -2 | -1 | | -2 | | 0 | | -2 | | 0 | -2 | | | 0 | | 0 | | 0 | | 0 | 0 | | | 0 | | | 0 | | 0 | | 0 | 510 | | 18 | | 3.5 | | 79 | | 6 | | 7.6 | OR | | 2.25 | | 0.86–5.84 | |
| Naoum, G.E. 2020 | Retrospective cohort | | -2 | -2 | | -2 | | 0 | | -2 | | 0 | -2 | | | 0 | | 0 | | 0 | | 0 | -1 | | | 0 | | | 0 | | 0 | | 0 | 603 | | 121 | | 20.1 | | 236 | | 89 | | 37.7 | OR | | 2.41 | | 1.73–3.36 | |
| Sewart, E. 2020 | Prospective cohort | | -2 | -2 | | -2 | | -2 | | -2 | | 0 | -2 | | | 0 | | 0 | | 0 | | 0 | 0 | | | 0 | | | 0 | | -2 | | -1 | 516 | | 26 | | 5.0 | | 214 | | 13 | | 6.1 | OR | | 1.22 | | 0.61–2.42 | |
| Chen, J.J. 2021 | Retrospective cohort | | -2 | -1 | | -2 | | 0 | | -2 | | 0 | -1 | | | 0 | | 0 | | 0 | | 0 | 0 | | | 0 | | | 0 | | 0 | | 0 | 30 | | 3 | | 10.0 | | 38 | | 8 | | 21.1 | OR | | 2.4 | | 0.58–9.98 | |
| Comment | |  |  | |  | | | |  | |  | | |  | | | |  | | |  | | |  | | | |  | | | |  | |  | |  | |  | |  | |  | |  |  | |  | |  | |
| Drucker-Zertuche, M. 2011 |  | | Differences in TNM stage were expected. | Difference in chemotherapy and endocrine therapy were expected. | | Unblinded | |  | | No adjustment for confounding was performed. | |  |  | | |  | |  | |  | |  |  | | |  | | |  | |  | |  |  | |  | |  | |  | |  | |  |  | |  | |  | |
| Jimenez-Puente, A. 2011 |  | | TNM status unknown. | Chemotherapy status unknown | | Unblinded | |  | | No TNM adjustment was performed in the multivariate analysis. | |  | The main object is not to compare the adverse events of irradiated and non-irradiated patients. | | |  | |  | |  | |  |  | | |  | | |  | |  | |  |  | |  | |  | |  | |  | |  |  | |  | |  | |
| Lin KY, 2011 |  | | TNM status unknown. | Difference in chemotherapy. | | Unblinded | |  | | No TNM adjustment was performed in the multivariate analysis. | |  |  | | |  | |  | |  | |  |  | | |  | | |  | |  | |  |  | |  | |  | |  | |  | |  |  | |  | |  | |
| Nava, M.B. 2011 |  | | Differences in TNM stage were expected. | Difference in chemotherapy and endocrine therapy were expected. | | Unblinded | |  | | Some confounding factors were adjusted. | |  |  | | |  | |  | |  | |  |  | | |  | | |  | |  | |  |  | |  | |  | |  | |  | |  |  | |  | |  | |
| Cordeiro, P.G. 2014 |  | | Differences in TNM stage were expected. | Difference in chemotherapy and endocrine therapy were expected. | | Unblinded | |  | | No adjustment for confounding was performed. | |  |  | | |  | |  | |  | |  |  | | |  | | |  | |  | |  |  | |  | |  | |  | |  | |  |  | |  | |  | |
| Anker, C.J. 2015 |  | | Difference in TNM stage. | Difference in chemotherapy. | | Unblinded | |  | | No adjustment for confounding was performed. | |  |  | | |  | |  | |  | |  |  | | |  | | |  | |  | |  |  | |  | |  | |  | |  | |  |  | |  | |  | |
| Kearney, A.M. 2015 |  | | Differences in TNM stage were expected. | Difference in chemotherapy. | | Unblinded | |  | | No adjustment for confounding was performed. | |  |  | | |  | |  | |  | |  |  | | |  | | |  | |  | |  |  | |  | |  | |  | |  | |  |  | |  | |  | |
| Matsukata, A. 2016 |  | | Differences in TNM stage were expected. | Difference in chemotherapy and endocrine therapy were expected. | | Unblinded | |  | | No adjustment for confounding was performed. | |  |  | | |  | |  | |  | |  |  | | |  | | |  | | Cases that did not result in implant reconstruction were classified as reconstruction failures. | |  |  | |  | |  | |  | |  | |  |  | |  | |  | |
| Lam, T.C. 2018 |  | | TNM status unknown. | Difference in chemotherapy. | | Unblinded | |  | | No correction in multivariate analysis. | |  |  | | |  | |  | |  | |  |  | | |  | | |  | |  | |  |  | |  | |  | |  | |  | |  |  | |  | |  | |
| Sinnott, C.J. 2018 |  | | TNM status unknown. | Chemotherapy status unknown | | Unblinded | |  | | No correction in multivariate analysis. | |  |  | | |  | |  | |  | |  |  | | |  | | |  | |  | |  |  | |  | |  | |  | |  | |  |  | |  | |  | |
| Naoum, G.E. 2020 |  | | More advanced stage in PMRT group | Difference in chemotherapy. | | Unblinded | |  | | No TNM adjustment was performed in the multivariate analysis. | |  |  | | |  | |  | |  | |  | Some delayed reconstruction was included. | | |  | | |  | |  | |  |  | |  | |  | |  | |  | |  |  | |  | |  | |
| Sewart, E. 2020 |  | | Large size in PMRT group | More patients received chemotherapy in PMRT group. | | Unblinded | | Short follow-up duration (3 months) | | No correction in multivariate analysis. | |  |  | | |  | |  | |  | |  |  | | |  | | |  | | Results within 3 months only | |  |  | |  | |  | |  | |  | |  |  | |  | |  | |
| Chen, J.J. 2021 |  | | TNM status unknown. | Chemotherapy status unknown | | Unblinded | |  | | No TNM adjustment was performed in the multivariate analysis. | |  |  | | |  | |  | |  | |  |  | | |  | | |  | |  | |  |  | |  | |  | |  | |  | |  |  | |  | |  | |

Supplementary Table 4. Risk of bias assessment for capsular contracture

| Outcomes | | | Capsular contracture with Baker grade III or IV and/or requiring additional surgery | | | | | | | | |  | |  |  | | |  | | |  |  | |  | |  | | |  | |  | | |  |  | |  |  | |  | |  | | |  | |  | |  | | |
| --- | --- | --- | --- | --- | --- | --- | --- | --- | --- | --- | --- | --- | --- | --- | --- | --- | --- | --- | --- | --- | --- | --- | --- | --- | --- | --- | --- | --- | --- | --- | --- | --- | --- | --- | --- | --- | --- | --- | --- | --- | --- | --- | --- | --- | --- | --- | --- | --- | --- | --- | --- |
| Individual study | | | Risk of bias | | | | | | | | |  | |  |  | | |  | | |  |  | |  | |  | | |  | |  | | |  |  | |  |  | |  | |  | | |  | |  | |  | | |
|  |  |  | Selection bias | | Performance bias | Detection bias | Attrition bias | | Others | | |  | | Factors that can increase the certainty of the evidence | | | | | | |  | Indirectness | | | | | | | | | | |  | Number at risk (outcome rate) | | | | | | | | | | |  |  | |  | | |  |
| Study code | Study design | | Differences between baseline characteristics | | Differences between groups in the care | Differences between groups in how outcomes are determined | Differences between groups in withdrawals | | Inadequate adjustment for confounding | Others | | Summary | | Dose-response gradient | | Effect of potential residual confounding factors | | | Large magnitude of effect | Summary | | Participants | | | Intervention | | | Comparison | | Outcome | | | Summary | The denominator for comparison arm | | The numerator for comparison arm | | | (%) | | The denominator for intervention arm | | The numerator for intervention arm | (%) | Effect measures | | An estimate of effect | | | 95% Confidence interval |  |
| Behranwala, K.A. 2006 | Retrospective cohort | | -2 | | -1 | -2 | 0 | | 0 | 0 | | -1 | | 0 | | 0 | | | 0 | 0 | | 0 | | | 0 | | | 0 | | 0 | | | 0 | 92 | | 2 | | | 2.2 | | 44 | | 10 | 22.7 | OR | | 13.24 | | | 2.76–63.53 |  |
| Whitfield, G.A. 2008 | Retrospective cohort | | -2 | | -1 | -2 | 0 | | -2 | 0 | | -1 | | 0 | | 0 | | | 0 | 0 | | 0 | | | 0 | | | 0 | | 0 | | | 0 | 78 | | 0 | | | 0.0 | | 42 | | 8 | 19.0 | OR | | 38.68 | | | 2.17–689.16 |  |
| Patani, N. 2008 | Retrospective cohort | | -2 | | -1 | -2 | 0 | | -2 | 0 | | -1 | | 0 | | 0 | | | 0 | 0 | | -1 | | | 0 | | | 0 | | 0 | | | 0 | 67 | | 9 | | | 13.4 | | 16 | | 14 | 87.5 | OR | | 45.11 | | | 8.75–232.45 |  |
| Drucker-Zertuche, M. 2011 | Retrospective cohort | | -2 | | -1 | -2 | 0 | | -2 | 0 | | -1 | | 0 | | 0 | | | 0 | 0 | | 0 | | | 0 | | | 0 | | 0 | | | 0 | 60 | | 2 | | | 3.3 | | 37 | | 8 | 21.6 | OR | | 8 | | | 1.60–40.12 |  |
| Nava, M.B. 2011 | Case-control | | -2 | | -1 | -2 | 0 | | -1 | 0 | | -1 | | 0 | | 0 | | | 0 | 0 | | 0 | | | 0 | | | 0 | | 0 | | | 0 | 91 | | 22 | | | 24.2 | | 121 | | 78 | 64.5 | OR | | 5.69 | | | 3.10–10.44 |  |
| Cordeiro, P.G. 2014 | Retrospective cohort | | -2 | | -1 | -2 | 0 | | -2 | 0 | | -1 | | 0 | | 0 | | | 0 | 0 | | 0 | | | 0 | | | 0 | | 0 | | | 0 | 1814 | | 116 | | | 6.4 | | 319 | | 147 | 46.1 | OR | | 12.51 | | | 9.37–16.71 |  |
| Pompei, S. 2017 | Retrospective cohort | | -2 | | -1 | -2 | 0 | | -2 | 0 | | -1 | | 0 | | 0 | | | 0 | 0 | | 0 | | | 0 | | | 0 | | 0 | | | 0 | 59 | | 3 | | | 5.1 | | 56 | | 6 | 10.7 | OR | | 2.24 | | | 0.53–9.43 |  |
| Elswick, S.M. 2018 | Retrospective cohort | | -2 | | -1 | -2 | 0 | | -2 | 0 | | -1 | | 0 | | 0 | | | 0 | 0 | | 0 | | | 0 | | | 0 | | 0 | | | 0 | 39 | | 0 | | | 0.0 | | 54 | | 1 | 1.9 | OR | | 2.21 | | | 0.09–55.82 |  |
| Hamann, M. 2019 | Retrospective cohort | | -2 | | -2 | -2 | 0 | | -2 | 0 | | -2 | | 0 | | 0 | | | 0 | 0 | | 0 | | | 0 | | | 0 | | 0 | | | 0 | 63 | | 1 | | | 1.6 | | 26 | | 6 | 23.1 | OR | | 18.6 | | | 2.11–163.91 |  |
| Comment | |  | |  | |  | |  | | |  | |  | | | |  | | | |  | |  | | | |  | | | | |  | |  | |  | | |  | |  | |  |  |  | |  | | |  |  |
| Behranwala, K.A. 2006 |  | | TNM status unknown. | | Chemotherapy status unknown | Unblinded |  | |  |  | |  | |  | |  | | |  |  | |  | | |  | | |  | |  | | |  |  | |  | | |  | |  | |  |  |  | |  | | |  |  |
| Whitfield, G.A. 2008 |  | | TNM status unknown. | | Chemotherapy status unknown | Unblinded |  | | No correction in multivariate analysis. |  | |  | |  | |  | | |  |  | |  | | |  | | |  | |  | | |  |  | |  | | |  | |  | |  |  |  | |  | | |  |  |
| Patani, N. 2008 |  | | TNM status unknown. | | Chemotherapy status unknown | Unblinded |  | | No correction in multivariate analysis. |  | |  | |  | |  | | |  |  | | Some pre-radiotherapy was included. | | |  | | |  | |  | | |  |  | |  | | |  | |  | |  |  |  | |  | | |  |  |
| Drucker-Zertuche, M. 2011 |  | | Differences in TNM stage were expected. | | Difference in chemotherapy and endocrine therapy were expected. | Unblinded |  | | No adjustment for confounding was performed. |  | |  | |  | |  | | |  |  | |  | | |  | | |  | |  | | |  |  | |  | | |  | |  | |  |  |  | |  | | |  |  |
| Nava, M.B. 2011 |  | | Differences in TNM stage were expected. | | Difference in chemotherapy and endocrine therapy were expected. | Unblinded |  | | Some confounding factors were adjusted. |  | |  | |  | |  | | |  |  | |  | | |  | | |  | |  | | |  |  | |  | | |  | |  | |  |  |  | |  | | |  |  |
| Cordeiro, P.G. 2014 |  | | Differences in TNM stage were expected. | | Difference in chemotherapy and endocrine therapy were expected. | Unblinded |  | | No adjustment for confounding was performed. |  | |  | |  | |  | | |  |  | |  | | |  | | |  | |  | | |  |  | |  | | |  | |  | |  |  |  | |  | | |  |  |
| Pompei, S. 2017 |  | | TNM status unknown. | | Chemotherapy status unknown | Unblinded |  | | No adjustment was performed in the multivariate analysis. |  | |  | |  | |  | | |  |  | |  | | |  | | |  | |  | | |  |  | |  | | |  | |  | |  |  |  | |  | | |  |  |
| Elswick, S.M. 2018 |  | | Differences in TNM stage were expected. | | Difference in chemotherapy and endocrine therapy were expected. | Unblinded |  | | No adjustment for confounding was performed. |  | |  | |  | |  | | |  |  | |  | | |  | | |  | |  | | |  |  | |  | | |  | |  | |  |  |  | |  | | |  |  |
| Hamann, M. 2019 |  | | More advanced stage in PMRT group | | Difference in chemotherapy. | Unblinded |  | | No adjustment was performed in the multivariate analysis. |  | |  | |  | |  | | |  |  | |  | | |  | | |  | |  | | |  |  | |  | | |  | |  | |  |  |  | |  | | |  |  |

Supplementary Table 5. Risk of bias assessment for cosmesis

| Outcomes | | Decline in cosmesis (less than "good") | | | | | | | | | |  | |  |  | | |  | | |  |  | | |  | |  | | |  | | |  | |  |  |  | |  | |  | |  | | |  |  | | | | | |  | | | | |
| --- | --- | --- | --- | --- | --- | --- | --- | --- | --- | --- | --- | --- | --- | --- | --- | --- | --- | --- | --- | --- | --- | --- | --- | --- | --- | --- | --- | --- | --- | --- | --- | --- | --- | --- | --- | --- | --- | --- | --- | --- | --- | --- | --- | --- | --- | --- | --- | --- | --- | --- | --- | --- | --- | --- | --- | --- | --- |
| Individual study | | Risk of bias | | | | | | | | | |  | |  |  | | |  | | |  |  | | |  | |  | | |  | | |  | |  |  |  | |  | |  | |  | | |  | | | |  |  | | | | | |  |
|  |  | Selection bias | | Performance bias | | Detection bias | Attrition bias | | Others | | |  | | Factors that can increase the certainty of the evidence | | | | | | |  | Indirectness | | | | | | | | | | |  | Number at risk (outcome rate) | | | | | | | | | | | | | |  |  | | | | | |  |  |  |
| Study code | Study design | Differences between baseline characteristics | | Differences between groups in the care | | Differences between groups in how outcomes are determined | Differences between groups in withdrawals | | Inadequate adjustment for confounding | | Others | Summary | | Dose-response gradient | | Effect of potential residual confounding factors | | | Large magnitude of effect | | Summary | Participants | | | | Intervention | | | Comparison | | Outcome | | Summary | The denominator for comparison arm | | | | The numerator for comparison arm | | (%) | | The denominator for intervention arm | | The numerator for intervention arm | (%) | | | Effect measures | | | | An estimate of effect | | 95% Confidence interval | |  |  |
| Drucker-Zertuche, M. 2011 | Retrospective cohort | -2 | | -1 | | -2 | 0 | | -2 | | 0 | -1 | | 0 | | 0 | | | 0 | | 0 | | 0 | | | 0 | | | 0 | | 0 | | 0 | 60 | | | | 2 | | 3.33 | | 36 | | 23 | 63.89 | | | OR | | | | 51.31 | | 10.73–245.41 | |  |  |
| Nava, M.B. 2011 | Case-control | -2 | | -1 | | -2 | 0 | | -1 | | 0 | -1 | | 0 | | 0 | | | 0 | | 0 | | 0 | | | 0 | | | 0 | | 0 | | 0 | 91 | | | | 29 | | 31.87 | | 116 | | 57 | 49.14 | | | OR | | | | 2.07 | | 1.17–3.66 | |  |  |
| Cordeiro, P.G. 2014 | Retrospective cohort | -2 | | -1 | | -2 | 0 | | -2 | | 0 | -1 | | 0 | | 0 | | | 0 | | 0 | | 0 | | | 0 | | | 0 | | 0 | | 0 | 1784 | | | | 64 | | 3.59 | | 279 | | 34 | 12.19 | | | OR | | | | 3.73 | | 2.41–5.77 | |  |  |
| Anker, C.J. 2015 | Retrospective cohort | -2 | | -2 | | -2 | 0 | | -2 | | 0 | -2 | | 0 | | 0 | | | 0 | | 0 | | 0 | | | 0 | | | 0 | | 0 | | 0 | 110 | | | | 41 | | 37.27 | | 39 | | 15 | 38.46 | | | OR | | | | 1.05 | | 0.50–2.23 | |  |  |
| Lam, T.C. 2018 | Retrospective cohort | -2 | | -2 | | -2 | 0 | | -2 | | 0 | -2 | | 0 | | 0 | | | 0 | | 0 | | 0 | | | 0 | | | 0 | | 0 | | 0 | 338 | | | | 36 | | 10.65 | | 114 | | 49 | 42.98 | | | OR | | | | 6.32 | | 3.81–10.50 | |  |  |
| Hamann, M. 2019 | Retrospective cohort | -2 | | -2 | | -2 | 0 | | -2 | | 0 | -2 | | 0 | | 0 | | | 0 | | 0 | | 0 | | | 0 | | | 0 | | 0 | | 0 | 63 | | | | 16 | | 25.40 | | 26 | | 11 | 42.31 | | | OR | | | | 2.15 | | 0.82–5.64 | |  |  |
| Comment |  | |  | |  | | |  | |  | | |  | | | |  | | |  | | | |  | | | |  | | | |  | |  | | | |  | |  | |  | |  |  | | |  | | | |  | |  | |  |  |
| Drucker-Zertuche, M. 2011 |  | Differences in TNM stage were expected. | | Difference in chemotherapy and endocrine therapy were expected. | | Unblinded |  | | No adjustment for confounding was performed. | |  |  | |  | |  | | |  | |  | |  | | |  | | |  | |  | |  |  | | | |  | |  | |  | |  |  | | |  | | | |  | |  | |  |  |
| Nava, M.B. 2011 |  | Differences in TNM stage were expected. | | Difference in chemotherapy and endocrine therapy were expected. | | Unblinded |  | | Some confounding factors were adjusted. | |  |  | |  | |  | | |  | |  | |  | | |  | | |  | |  | |  |  | | | |  | |  | |  | |  |  | | |  | | | |  | |  | |  |  |
| Cordeiro, P.G. 2014 |  | Differences in TNM stage were expected. | | Difference in chemotherapy and endocrine therapy were expected. | | Unblinded |  | | No adjustment for confounding was performed. | |  |  | |  | |  | | |  | |  | |  | | |  | | |  | |  | |  |  | | | |  | |  | |  | |  |  | | |  | | | |  | |  | |  |  |
| Anker, C.J. 2015 |  | Difference in TNM status. | | Difference in chemotherapy. | | Unblinded |  | | No adjustment for confounding was performed. | |  |  | |  | |  | | |  | |  | |  | | |  | | |  | |  | |  |  | | | |  | |  | |  | |  |  | | |  | | | |  | |  | |  |  |
| Lam, T.C. 2018 |  | TNM status unknown. | | Difference in chemotherapy. | | Unblinded |  | | No adjustment was performed in the multivariate analysis. | |  |  | |  | |  | | |  | |  | |  | | |  | | |  | |  | |  |  | | | |  | |  | |  | |  |  | | |  | | | |  | |  | |  |  |
| Hamann, M. 2019 |  | Advanced stage in PMRT group | | Difference in chemotherapy. | | Unblinded |  | | No adjustment was performed in the multivariate analysis. | |  |  | |  | |  | | |  | |  | |  | | |  | | |  | |  | |  |  | | | |  | |  | |  | |  |  | | |  | | | |  | |  | |  |  |

Supplementary Table 6. Body of evidence by each outcome

|  |  |  |  |  |  |  |  | Number at risk (outcome rate) | | | | | |  |  |  |  |  |
| --- | --- | --- | --- | --- | --- | --- | --- | --- | --- | --- | --- | --- | --- | --- | --- | --- | --- | --- |
| Outcomes | Study design /Number of studies | Risk of bias | Inconsistency | Imprecision | Indirectness | Others (Publication bias) | Factors that can increase the certainty of the evidence | The denominator for comparison arm | The numerator for comparison arm | (％) | The denominator for intervention arm | The numerator for intervention arm | (％) | Effect measures | An estimate of effect | 95% Confidence interval | Certainty of evidence | Importance |
| Major complications | Cohort /11 | -1 | -1 | 0 | 0 | -1 | 0 | 3654 | 350 | 9.6 | 996 | 211 | 21.2 | OR | 2.62 | 1.82–3.77 | Low (C) | 8 |
| Reconstruction failure | Cohort /12,  Case-control /1 | -1 | -1 | -1 | 0 | 0 | 0 | 4713 | 265 | 5.6 | 1366 | 236 | 17.3 | OR | 3.32 | 2.02–5.45 | Low (C) | 8 |
| Capsular contracture | Cohort /9,  Case-control /1 | -1 | 0 | -1 | 0 | 0 | 0 | 2436 | 155 | 6.4 | 742 | 280 | 37.7 | OR | 9.63 | 5.77–16.06 | Low (C) | 8 |
| Decline in cosmesis | Cohort /5,  Case-control /1 | -1 | -1 | 0 | 0 | 0 | 0 | 2446 | 188 | 7.7 | 611 | 189 | 30.9 | OR | 3.55 | 1.80–6.98 | Low (C) | 7 |


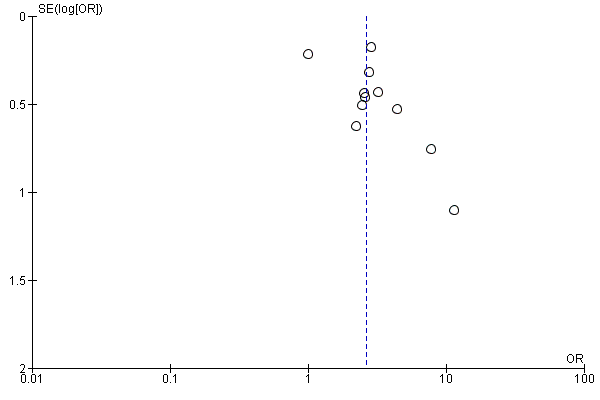


Supplementary Figure 1. Funnel plot evaluating publication bias for major complications


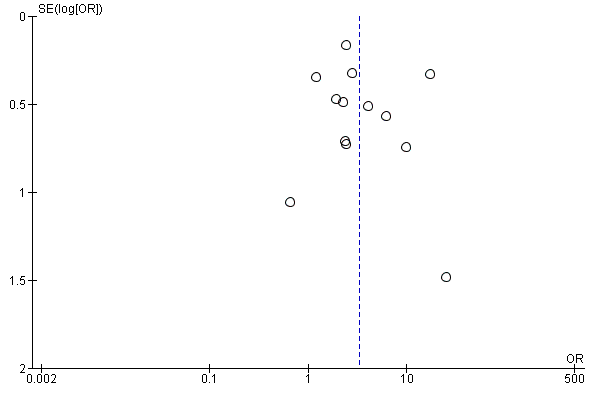


Supplementary Figure 2. Funnel plot evaluating publication bias for reconstruction failure


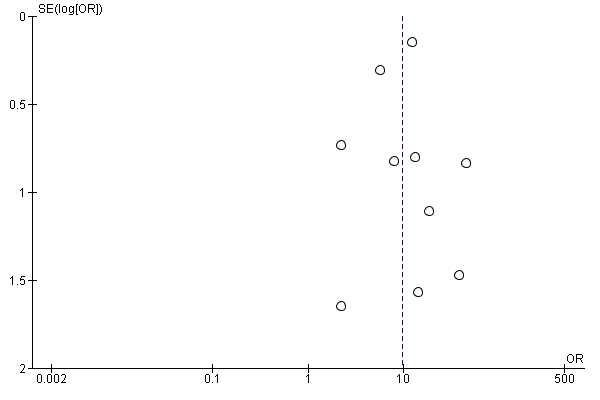


Supplementary Figure 3. Funnel plot evaluating publication bias for capsular contracture


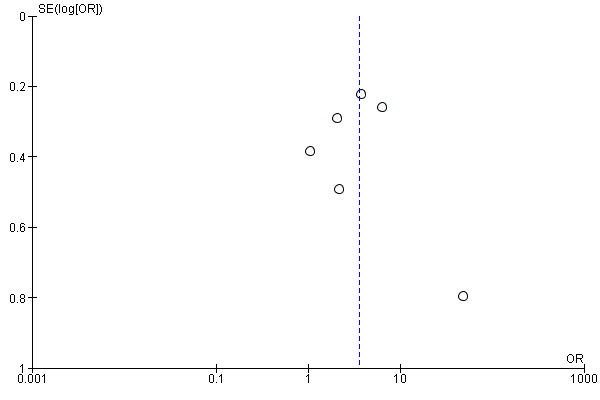


Supplementary Figure 4. Funnel plot evaluating publication bias for cosmesis
